# Supplementary material for: A Novel Major Output Target for Pheromone-Sensitive Projection Neurons in Male Moths
Source: Front Cell Neurosci. 2020 Jun 8;14:147. doi: 10.3389/fncel.2020.00147 (PMC7294775; doi:10.3389/fncel.2020.00147)
Supplement: Supplementary file 1 [file Data_Sheet_1.DOCX]

Supplementary Material

# Supplementary Figures and Tables

## Supplementary Figures


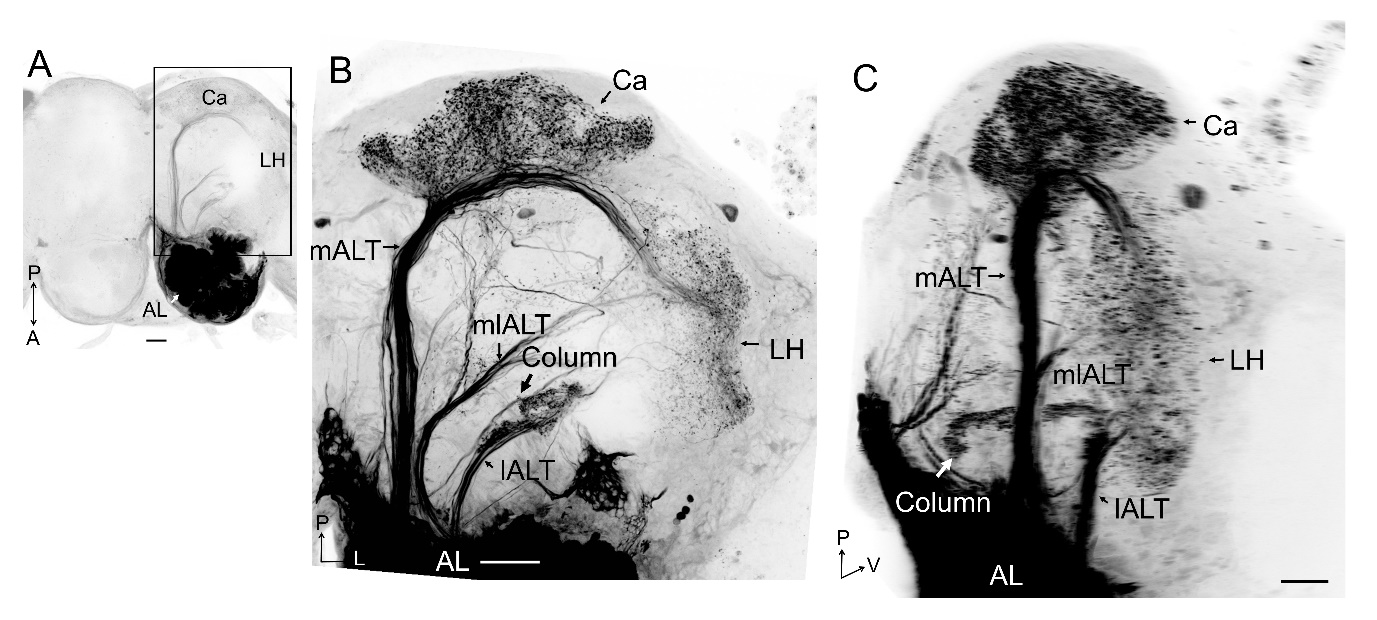


**Fig. S1 | Projection profiles of antennal lobe** (AL) output neurons in female. (**A**) Confocal image of the left AL illustrating the ‘anterograde labeling’ sites in the AL. (**B-C**) Confocal images of the mass-stained preparation showing the three main AL tracts (ALTs) in dorsal view (**B**) and sagittal view (**C**). The strongly labeled column indicates that this area is a main target of lateral-tract neurons in the female moth. However, no commissural fiber bundle originating from the AL was visible here. (l/m/ml)ALT, (lateral/medial/ mediolateral) antennal lobe tract; Ca, Calyces of the mushroom body; LH, lateral horn; A, anterior; L, lateral; M, medial; P, posterior; V, ventral. Scale bars, 50 μm.


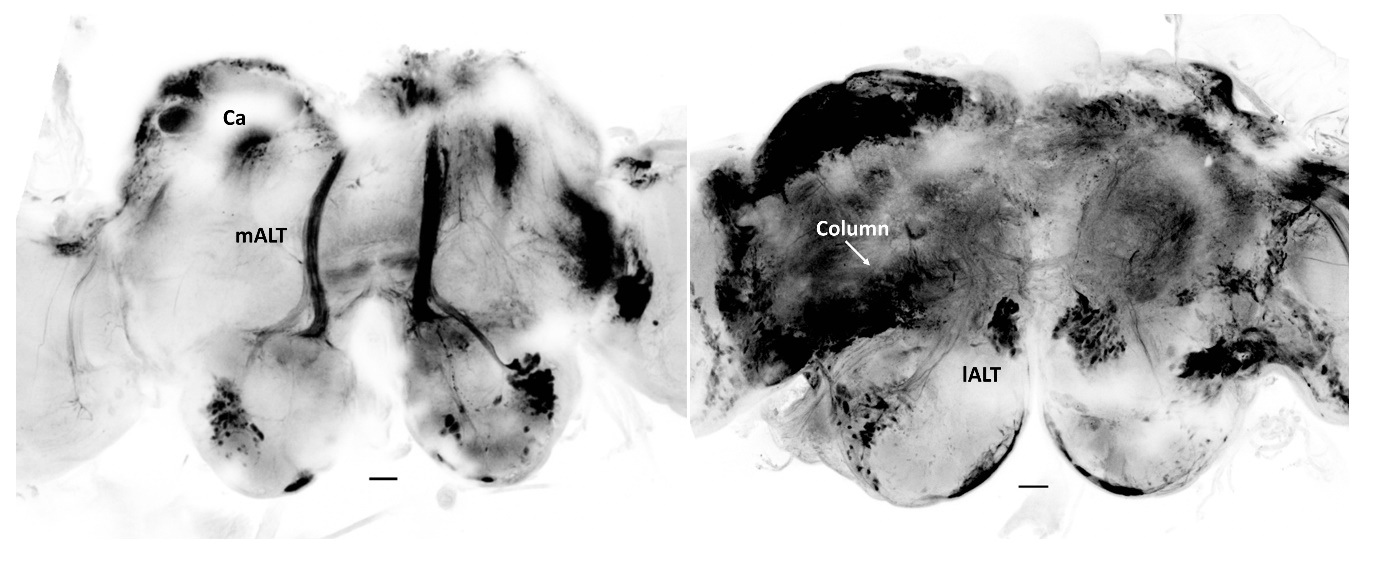


**Fig. S2 | Selective retrograde labeling of projection neurons in preparations used for calcium imaging experiment (dorsal view).** ***Left***: exclusive labeling of medial-tract neurons when the mixture of Fura-2 and AF488 was applied into the calyces (Ca). ***Right***: labeling of lateral-tract neurons when we the dye mixture was applied into the superior intermediate protocerebrum (SIP). (l/m)ALT, (lateral/medial) antennal lobe tract; Scale bars, 50 μm.


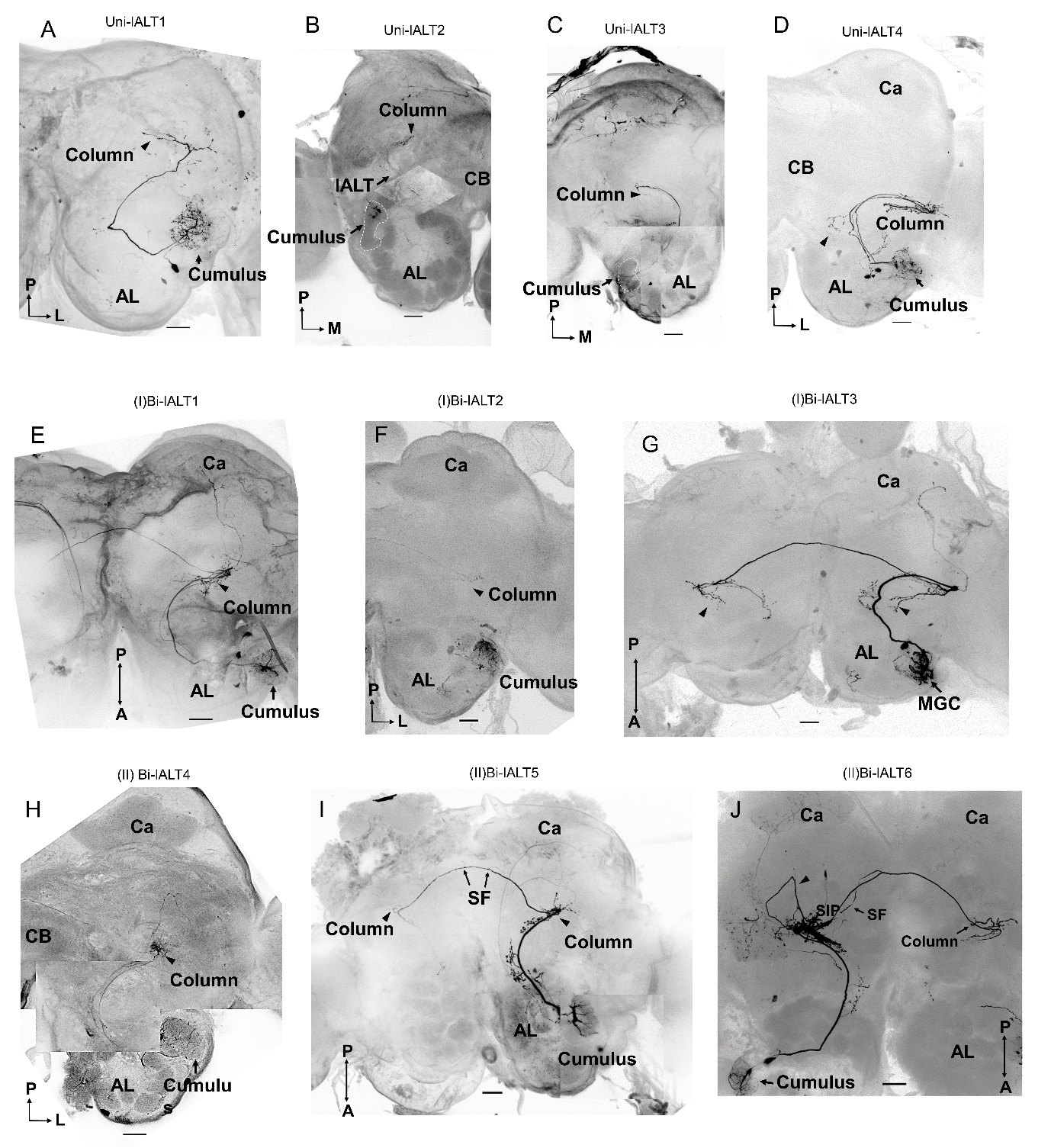


**Fig. S3 |** **Morphologies of all ten recorded lateral-tract neurons**. (**A-D**) Confocal images of the four unilateral lateral-tract neurons. (**E-G**) Confocal images of three Sub-type I bilateral lateral‑tract neurons. (**H-J**) Confocal images of three Sub-type II bilateral lateral‑tract neurons. AL, antennal lobe; Ca, Calyces of the mushroom body; CB, central body. A, anterior; L, lateral; M, medial; P, posterior; V, ventral. Scale bars, 50 μm.


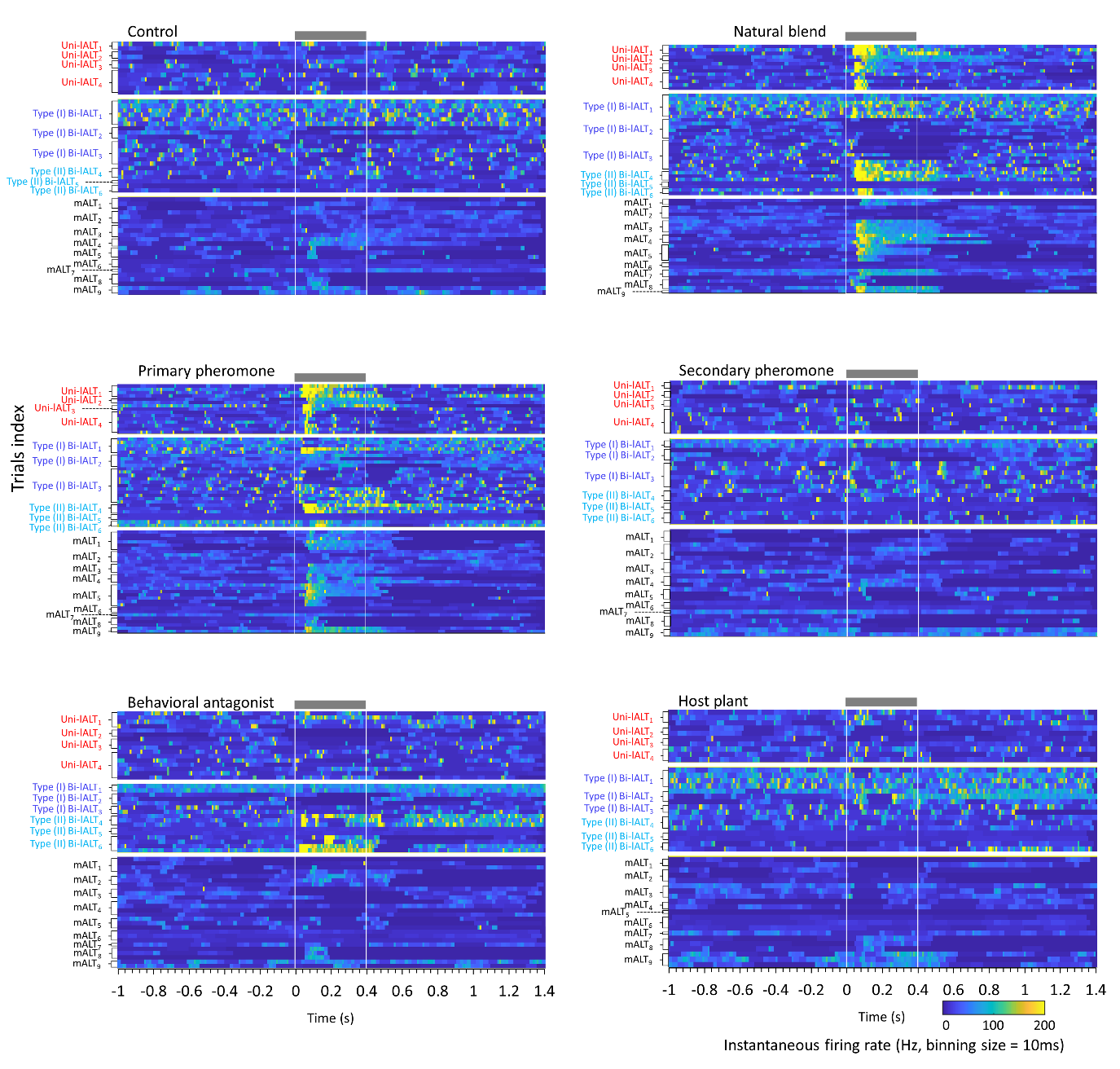


**Fig. S4 | Neuron spiking activities of the individual antennal-lobe output neurons during application of pheromone and plant odor stimuli.** Responding profiles can be seen for ten lateral‑tract cumulus neurons and nine medial‑tract cumulus neurons, illustrated by the instantaneous firing rate heat map. Each row plots an individual trial. The *grey* bar represents the onset and duration of the stimulus (400ms).


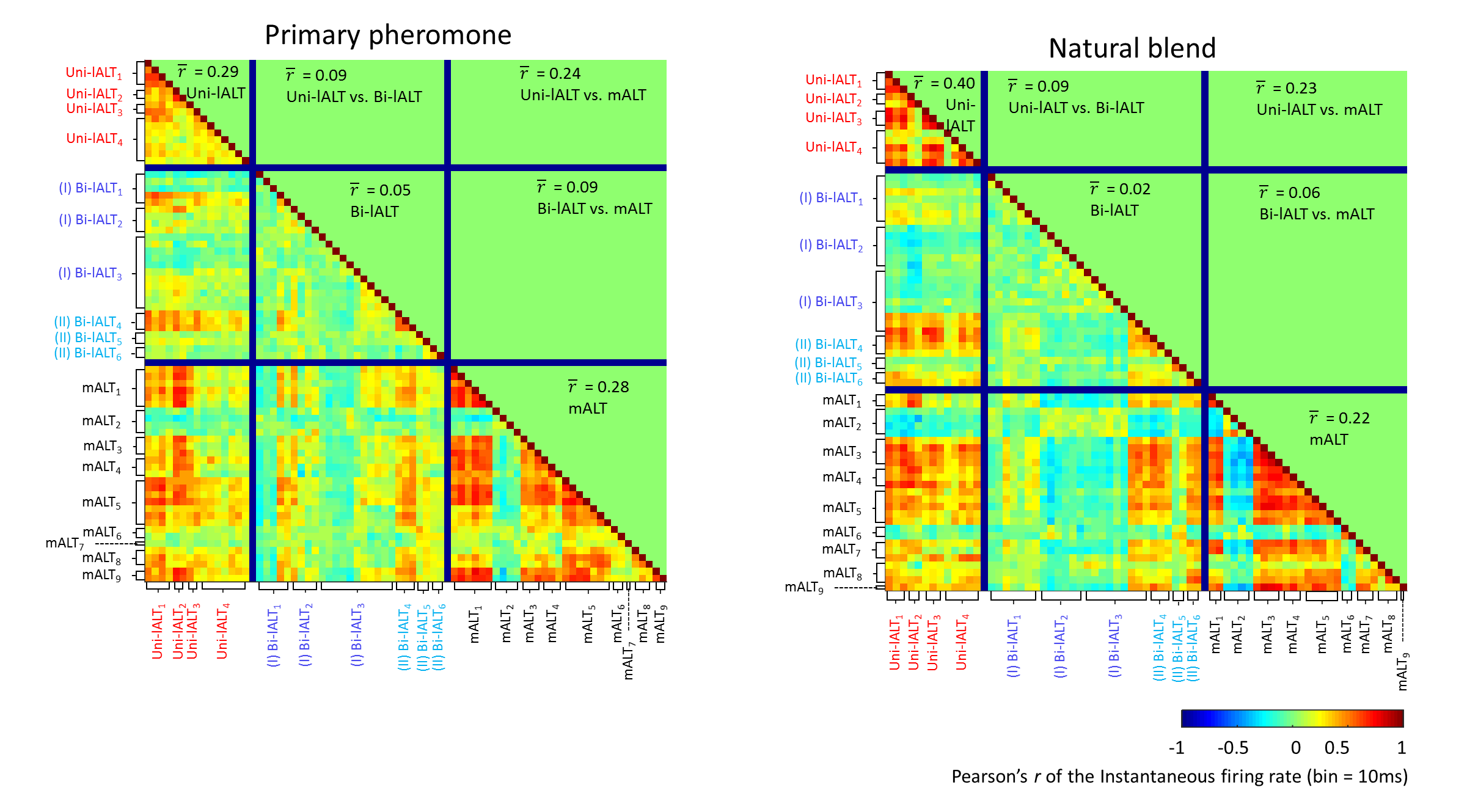


**Fig. S5 | Pairwise correlation plots of the instantaneous firing rate (IFR, bin-size: 10 ms) to the primary pheromone (*left*) and natural blend (*right*).** Each row plots the pearson’s *r* according to the IFRs of every two trials either within the same neuron type or across two different types. The mean correlation values ($\overline{r}$) of neurons within each type or between two types are presented within the corresponding area located symmetric along the diagonal.


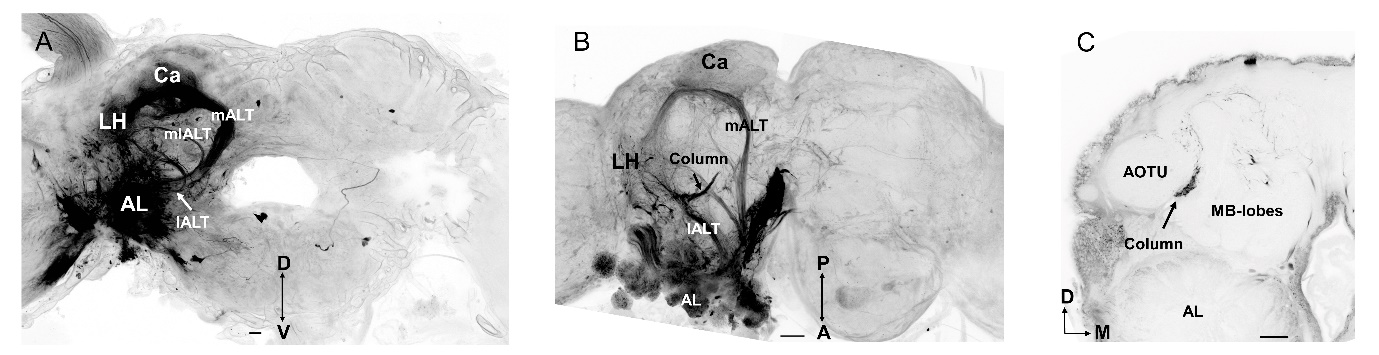


**Fig. S6 | Projection profiles of antennal-lobe output neurons in other moth species.** (**A**) Lateral‑tract neurons in the Chinese oak silk moth, *Antheraea pernyi,* project to the lateral horn. (**B-C**) The column is the main target region of neurons confined to the lateral tract in the Silver Y, *Autographa gamma* (**B**) and Bogong moth, *Agrotis infusa* (**C**). Scale bars, 50 μm.


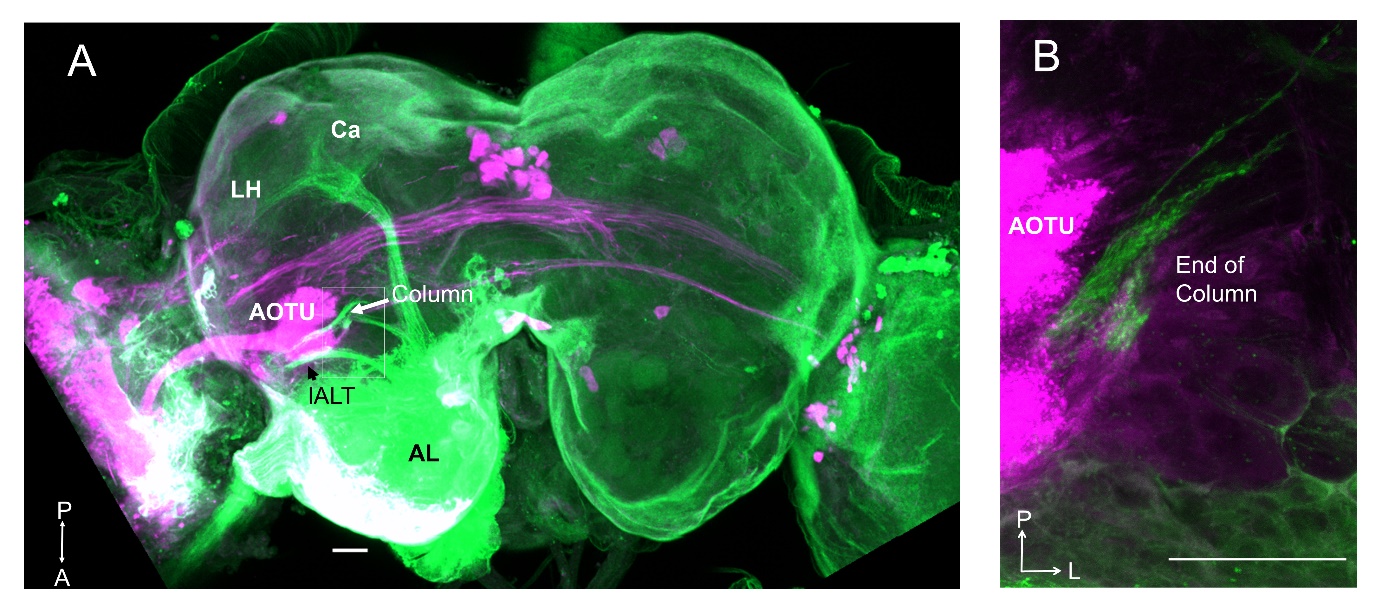


**Fig. S7 | The target of antennal-lobe (AL) output neurons passing along the lateral tract is not overlapping with the prominent visual neuropil, the anterior optic tubercle (AOTU).** (**A**) Confocal image in dorsal view of a double-labeled brain where different dyes were applied to the right optical lobe (*magenta*) and right antennal lobe (AL, *green*). (**B**) Magnified image of the area covered by the *white* square in (**A**), showing that the optic-lobe neurons projecting to the AOTU have no direct connection with the lateral-tract AL neurons. Scale bars, 50 μm.

**Table S1.** **Overview of neuronal parameters during pre-test activity in recorded neurons confined to lateral tract and medial tract.**

$\bar{\mathbf{ISI}}$: mean interspike interval, $\bar{\mathbf{FR}}$: mean firing rate, **ISI C*v***: interspike interval coefficient of variation, **ISI_min_**: the minimum interspike interval.

| **Neuron ID** | $\overline{\mathbf{ISI}}$ (ms) | $\overline{\mathbf{FR}}$ (Hz) | **ISI *Cv*** | **ISI_min_** (ms) |
| --- | --- | --- | --- | --- |
| **Uni-lALT_1_** | 26.3 | 38.02 | 1.09 | 2.91 |
| **Uni-lALT_2_** | 49.32 | 20.28 | 1.42 | 9.25 |
| **Uni-lALT_3_** | 49.24 | 20.31 | 1.06 | 3.09 |
| **Uni-lALT_4_** | 37.81 | 26.45 | 0.98 | 2.74 |
| **(I) Bi-lALT_1_** | 11.83 | 84.5 | 0.26 | 6.29 |
| **(I) Bi-lALT_2_** | 26.92 | 37.14 | 0.79 | 6.7 |
| **(I) Bi-lALT_3_** | 29.19 | 34.26 | 1.14 | 2.28 |
| **(II) Bi-lALT_4_** | 78.83 | 12.69 | 0.95 | 2.55 |
| **(II) Bi-lALT_5_** | 93.52 | 10.69 | 0.12 | 68.64 |
| **(II) Bi-lALT_6_** | 37.43 | 26.72 | 0.73 | 4.87 |
| **mALT_1_** | 57.55 | 17.38 | 1.85 | 10.74 |
| **mALT_2_** | 35.27 | 28.36 | 0.82 | 10.66 |
| **mALT_3_** | 48.98 | 20.42 | 1.45 | 10.66 |
| **mALT_4_** | 75.36 | 13.27 | 1.71 | 10.42 |
| **mALT_5_** | 62.48 | 16.01 | 1.49 | 5.69 |
| **mALT_6_** | 117.7 | 8.5 | 0.98 | 41.14 |
| **mALT_7_** | 26.52 | 37.7 | 0.63 | 6.17 |
| **mALT_8_** | 87.88 | 11.38 | 1.17 | 8.81 |
| **mALT_9_** | 32.64 | 30.63 | 1.36 | 9.17 |
